# Addressing treatment fatigue, therapeutic inertia, and cognitive dissonance in musculoskeletal pain management in primary care setting

**DOI:** 10.3389/fmed.2025.1543434

**Published:** 2025-06-25

**Authors:** Waseem Jerjes, Daniel Harding, Aidan Hilton

**Affiliations:** ^1^Department of Primary Care and Public Health, Faculty of Medicine, Imperial College London, London, United Kingdom; ^2^North End Medical Centre, Hammersmith and Fulham Primary Care Network, London, United Kingdom

**Keywords:** treatment fatigue, therapeutic inertia, cognitive dissonance, primary care, musculoskeletal pain

## Introduction

Chronic musculoskeletal (MSK) pain represents a leading cause of reduced disability-adjusted life years worldwide, with a marked impact on quality of life and day-to-day functioning (1). Major common MSK conditions, including osteoarthritis, chronic lower back pain, chronic neck pain, and tendinopathy, all require long-term management. In the majority of chronic MSK conditions, primary care physicians (PCPs) have the responsibility for the continued and comprehensive care required (with the exception of rheumatological conditions), and 1 in 7 PCP consultations have been found to be for a MSK condition (2).

Treatment of chronic MSK pain goes beyond symptom management (3). PCPs face challenges to fully implementing evidence-based long-term strategies for managing chronic MSK pain. For instance, treatment fatigue, therapeutic inertia, and cognitive dissonance can contribute toward disengagement from care and result in suboptimal patient outcomes. These phenomena are interrelated, featuring self-reinforcing feedback loops that can introduce resistance to treatment.

Any response to counteract these problems should take a multilevel, integrative approach that combines clinical expertise, psychological insight, and systemic innovation. This opinion paper will discuss in detail the challenges in implementing efficacious care for chronic MSK pain, whilst offering evidence-based strategies that can be implemented into the primary care setting to improve patient outcomes. In this context, treatment fatigue refers to the physical and psychological exhaustion patients feel from prolonged and demanding treatment regimens. Therapeutic inertia reflects a clinical failure to adapt or escalate care despite clear need for change. Cognitive dissonance, affecting both patients and clinicians, involves psychological discomfort when beliefs conflict with actions or evidence—often leading to treatment resistance or inaction (1, 4, 5).

### Treatment fatigue in chronic MSK pain management

Management of long-term MSK conditions requires an organized model of treatment, with conservative first-line treatments such as patient education and exercise therapy before the application of NSAIDs, steroid injections, or psychotherapy (2, 3). Treatment phases defined by the clinician can facilitate better decision-making and prevent *ad-hoc* treatment selection (3). When these measures fail, referral to the orthopedic team for consideration of further interventions, including surgical, may be indicated. For many patients, the lack of full or immediate relief may trigger frustration and disengage them from the prescribed care plan.

Treatment fatigue can become a major barrier, particularly as many physiotherapeutic, psychotherapeutic, or lifestyle interventions offer only slow, incremental benefits (4). When patients perceive limited gains from therapy, their internal motivation to persist weakens. This can evolve into disengagement from care, which in turn reduces the opportunity for treatment to work, leading to a self-reinforcing spiral of frustration and passivity (6, 7).

This slower response is partly attributable to the complex interplay between nociplastic pain mechanisms and central sensitization, both of which are common in chronic MSK pain populations. Patients may present with psychological comorbidities such as depression or anxiety, lower self-efficacy, or pain catastrophising, all of which can reduce the effectiveness of behavioral and physical interventions. These individual factors can be identified using validated tools such as the STarT Back Screening Tool or the Örebro Musculoskeletal Pain Questionnaire, which help stratify risk and personalize care pathways. Better assessment using these tools can inform prognosis, guide appropriate referral, and improve the timing and selection of interventions to maintain patient engagement and avoid frustration (4, 6, 8).

Over time, patients may experience these as diminishing gains, which may subsequently reduce their motivation for continued engagement or active participation. This can be exacerbated in patients who come with an pre-existing biomedically-focused belief system about their chronic MSK condition, who may have poor knowledge about, or belief in the benefits of longer-term non-medical interventions (5, 9, 10). Long-term pharmacological treatment with enduring side effects, as well as cost considerations, generally results in treatment fatigue. Side effects of medication must be discussed openly by clinicians, cost-effective options considered, and non-pharmacological methods such as cognitive-behavioral therapy (CBT) added to enhance compliance and alleviate frustration (4, 5, 11).

Evidence exists that the implementation of structured patient education and motivational interviewing enhances compliance with treatment and prevents discouragement (6). These strategies help the patient reframe experiences and remain committed to long-term management (1, 6, 12). This care withdrawal may sometimes create a vicious cycle: reduced adherence to management that leads to poor outcomes, further persuading patients that their treatment has been ineffective.

PCPs need to address the burden of treatment fatigue in a patient-centered manner by combining structured goal-setting with shared decision-making. Clear, step-by-step treatment objectives have been found to cut disengagement and enhance long-term compliance (1, 6, 7, 9). This method allows the patient to feel a sense of accomplishment, minimizing the frustration of working with slowly-acting therapies. Use of shared decision-making has been shown to enhance perceived pain reduction, and treatment adherence, and conversely absence of shared-decision making has been associated with reduced reported function and quality of life, as well as perpetuating reliance on analgesia-seeking as a patient strategy (5, 10, 13–15). The ability of the patients to participate in developing their treatment plans—which includes stating their values, preferences, and concerns—can lessen the emotional load in long-term management. Clinicians must establish step-by-step, patient-centered targets that align with individual lifestyle characteristics (2, 6, 8, 13). Adherence to treatment is supported by goal-setting intervention by providing measurable targets that facilitate progress and reinforce motivation. Furthermore, clinicians should provide education around the benefits of non-medical interventions when this is lacking, or gently challenge unhelpful health beliefs, to ensure patients make informed choices. All successes, even those perceived as small, should be celebrated for the patient to remain motivated.

Complementary therapies such as acupuncture and hydrotherapy could complement evidence-based treatments to enhance patient compliance in selected patients should they accord with patient beliefs. Evidence suggests that if the treatment plans match the health beliefs of the patient, compliance and the efficacy of the treatment are enhanced (9, 14). This is in accordance with the overall goal of maintaining participation in chronic MSK pain management by ensuring a patient-centered treatment, and may represent an alternate approach in such patients which have reached treatment fatigue or disengagement with more conventional strategies (1, 5).

### The impact of therapeutic inertia in chronic MSK pain management

One of the most important barriers that must be overcome to effectively manage MSK pain is therapeutic inertia, defined as failure to escalate or adjust treatments in the face of a stagnant or inadequate response (7).

The causes of therapeutic inertia are complex and multifactorial, including the time constraints of consultations in primary care, the complexity of chronic diseases, and patient beliefs about potential side effects or lack of efficacy of newer therapies (6, 8, 9). Therapeutic inertia in chronic MSK pain can firstly manifest as current therapies being reviewed and maintained at ineffective levels, rather than progressing treatments (e.g. increasing analgesic dosages or better adapting physiotherapy exercises). There may also be an inherent cognitive bias contributing to this—both patients and clinicians can become accustomed to a certain level of suboptimal improvement, viewing it as perhaps the best possible outcome, combined with fear of actively introducing any new adverse effects from changing current treatments.

Secondly, therapeutic inertia may manifest as resistance to try newer evidence-based treatments, perhaps for similar reasons of bias, or also lack of clinician familiarity with their use. Additionally, there remains a critical training gap in musculoskeletal medicine during undergraduate and postgraduate education, particularly in areas such as pain neurophysiology, psychosocial approaches, and the use of modern digital tools or non-pharmacologic therapies. Many PCPs report limited confidence in escalating non-traditional interventions or managing multidisciplinary care plans. This educational deficit can reinforce reliance on familiar but outdated regimens, perpetuating therapeutic inertia and making it harder to adopt evidence-based innovations in chronic pain care (1, 5, 10, 13). Therefore, overcoming therapeutic inertia requires continuous professional development alongside openness on the part of both clinician and patient to ensuring usual therapies can be adapted or intensified, or at other times integrating newer evidence-based treatments into their clinical practice.

Before the application of more recent pharmacological therapies or neuromodulation techniques, a structured model of pain management should first be applied. Such a model positions conservative approaches such as patient education and behavioral intervention first, prior to the use of more complex treatments. If first-line treatment is not providing relief, treatments such as duloxetine, a serotonin-norepinephrine reuptake inhibitor, could subsequently be considered, as it is effective in the management of chronic musculoskeletal pain and reduces opioid dependency (9). Similarly, electrical neuromodulation has also been found to promote functional recovery by modulating the mechanisms of pain (14). Treatment reviews should be included in practice routinely to ensure that the intervention remains appropriate clinically and in accordance with current evidence-based practice guidance. It is important to distinguish therapeutic inertia from clinically appropriate restraint—where non-escalation reflects well-judged caution. In contrast, inertia involves unjustified stagnation in care due to habit, fear, or uncertainty (2, 8).

Another crucial element is the involvement of the multidisciplinary team. Targeted interactions on the advice of the PCP, with either pain specialists, physiotherapists, musculoskeletal specialists, and psychologists can help patients avoid falling into a pattern of stagnation and ensure that treatment plans are adaptive and responsive to a patient's evolving needs. Closer collaboration between these different professionals can be achieved for instance, through managing patients in integrated care pathways, or through regular multidisciplinary team meetings.

### Cognitive dissonance in chronic MSK pain management: clinician and patient perspectives

Cognitive dissonance—or the discomfort that arises from conflicting beliefs or information—can develop in both patients and their treating clinicians during the management of chronic MSK pain. Patients may unintentionally carry beliefs that go against evidence-based recommendations. For instance, some patients are initially scared that exercise will worsen their pain, despite the abundant evidence existing to support that activity optimizes treatment of conditions like chronic lower back pain and tendinopathy (6, 14). This resistance can manifest as non-compliance or avoidance of certain treatments, which delays progress. To extend this example, in primary care this may manifest as patients not self-referring to, or not attending appointments with physiotherapy, despite initial agreement to do so.

Clinicians may also be subjected to cognitive dissonance when new treatment methods clearly challenge their traditional ways of doing things. For example, despite increasing evidence supporting the superiority of non-opioid strategies in managing chronic pain, some clinicians are resistant to moving away from opioid prescriptions, especially when such medications have provided some relief for the patient or alternate medication is contraindicated (10). This dissonance may be reinforced by limited access to training updates or continuing professional development in chronic pain management. Without regular exposure to emerging evidence, clinicians may struggle to reconcile previous clinical habits with evolving best practice, especially if they lack institutional support or protected learning time (6, 10).

Additionally, they may see long-term analgesic options as an acceptable compromise, if the patient is resistant to accessing local psychotherapeutic or physiotherapeutic services. The risk here is that adherence to outdated practices can prevent patients from receiving safer and more effective treatment modalities.

Dealing with cognitive dissonance requires open communication and education (11). In the case of the patient, this may involve the clinician openly and non-judgmentally exploring resistance to certain therapies, as well as addressing specific patient concerns, such as reframing the pain concept by showing that movement and activity will not further injure them but help. Education and nuanced engagement with patient concerns forms a significant part of overcoming this psychological barrier to adherence. It goes without saying that such conversations need both time and an ongoing relationship with the patient, which can prove challenging in the current primary care landscape. Reflective practice is also key for clinicians in developing communication skills around this. Attending multidisciplinary meetings, participating in case discussions, and seeking continuing medical education may also help clinicians reconcile their beliefs with new evidence and thus improve their willingness to adopt an innovative approach. These dynamics can be framed using the COM-B model, which highlights that sustained health behavior change requires not just capability and motivation, but an environment that actively supports change—an element often missing in fragmented pain care systems (1, 4, 7, 14).

## Discussion

### Multidisciplinary approaches: a path to success

Multidisciplinary pain management teams will best operate as part of a structured pathway of care that specifies the team's function at different points of the treatment process and when specialist input is necessary to support the primary care team (14, 16, 17). MDTs ought not to be applied as a blanket policy but selectively introduced on the basis of prior clinical criteria with primary care as the central coordinating hub of care. In this way, unnecessary fragmentation is obviated and long-term MSK pain management is maximized by having a clear framework of when and how multidisciplinary intervention is to be utilized (1, 12). These teams may comprise PCPs, rheumatologists, pain specialists, orthopedic specialists, physiotherapists, psychologists, social prescribers, and nutritionists (6, 8). A more integrated approach considers not just physical but also psychological and emotional angles, not to mention lifestyle considerations as background issues for disengagement by the patient.

There also needs to be psychological intervention with the application of cognitive behavioral therapy (CBT), if feasible, in primary care to prevent unnecessary referral to specialist clinics. Patients would have improved tolerance to pain and improved compliance with rehabilitation programs by having psychological support initiated early. CBT helps patients to reframe their experience of pain by making them more resilient and engaged in the psychological and social effects of their condition (6).

Similarly, physiotherapists contribute by developing specific exercise programs which the patients are more likely to adhere to because they align with the patient's capabilities and recovery goals (6, 10).

The advantage of such a multidisciplinary approach is that treatment options can remain dynamic, in accordance with evolving needs of the patient. However, meaningful change also requires system-level reform. Time-limited consultations, lack of protected learning time, and siloed communication structures all contribute to the persistence of therapeutic inertia and disengagement. Addressing these structural barriers is essential if innovation at the clinical level is to succeed (3, 5, 6). Support by such a team—that addresses both physical and emotional needs—aids patients in their care engagement.

### Breaking down silos: enhancing care coordination

There is usually a lack of continuity in the transition between primary and specialist MSK pain services. To remedy this, referral pathways need to be established that provide a smooth transition, with initial management being managed by primary care doctors and specialist input coordinated as necessary. Lack of continuity often translates into inconsistent treatment plans, miscommunication, and lost opportunities to update care as the patient's condition evolves (2, 6, 15). For instance, a patient might receive physical therapy in one setting while continuing an outdated medication regimen prescribed in another.

Digital health technologies, including primary care-led virtual pain management platforms, can enhance communication between primary care and specialist services (18, 19). These platforms facilitate real-time collaboration, reducing reliance on isolated specialist referrals and ensuring a more coordinated approach. By streamlining care pathways, they minimize treatment delays and improve continuity of care for patients with chronic MSK pain that have not adequately responded to common treatments (5, 8, 19). Alongside reducing care delays, it also allows for immediate adjustments in treatment based on patient feedback and data.

Care can also be coordination-enhanced by establishing “pain management hubs” that bring pain management specialists from multiple disciplines together in one physical or virtual location. Primary care doctors should integrate multidisciplinary input via organized case discussion instead of multiple independent referrals (2, 7, 20). Such a system streamlines the delivery of care, eliminating the need for fragmented specialist visits and improving treatment continuity.

### The role of personalized medicine and digital health technologies

There is enormous potential in the future for overcoming the challenges in managing MSK pain through emergent technologies and personalized medicine (13). By tailoring treatment to the needs and genetic profiles of the individual, clinicians may one day be able to give more efficacious treatment while reducing side effects. Pharmacogenomic testing, for example, has the potential to one day allow clinicians to identify those people at greater risk of opioid dependence or persons metabolizing NSAIDs differentially and hence targeted medication regimens (17, 21, 22).

The integration of digital health technologies into daily life further expands the possibility for personalized treatment. Telemedicine allows patients, particularly with limited mobility, to have more flexibility in consulting services without necessarily having to travel far from home on a regular basis (2, 16). However, there has also been concern about whether telemedicine could make preserving therapeutic relationships more challenging, and about its unique barriers around digital literacy and broadband access (17).

Wearable devices are an emerging area of interest, and can allow patients to record data on factors including pain level tracking, physical activity levels, and sleep (23). This could help some patients more effectively manage their own day to day care, and there has been evidence that it can improve engagement in physical activity in patients with chronic MSK pain (18). The data these tools generate could potentially enrich clinician assessments, help make necessary changes in treatment plans, avoid therapeutic inertia, and address the first signs of treatment fatigue.

### Clinical relevance: real-life solutions for primary care

When suggesting solutions to improving chronic MSK management in primary care, a pragmatic approach is essential (10, 14, 18). To make reality of these ideas, one must consider how the suggestions can be achieved in an already overburdened healthcare setting without diverting crucial resources away from other patient groups.

For example, a key strategy is to facilitate patients to maintain their commitment to treatment by splitting complicated regimens of treatment into smaller and attainable steps, moving away from overwhelming, unattainable therapeutic goals. An example would be developing realistic, interim objectives such as regaining function or improving a specific functional element that gives the person a perception or satisfaction of achievement (19). This keeps them motivated and interested in their treatment. Such an approach may stave off treatment fatigue by providing more patient ownership over goal attainment, as well as highlighting incremental successes. Repeated evidence of gradual achievements may also help challenge the negative beliefs underpinning cognitive dissonance. Here telemedicine can be employed to deliver regular check-ups that can support and reassess patients with less downtime between cases (Table 1).

**Table 1:** Overcoming barriers in musculoskeletal pain management: challenges, solutions, and clinical examples.

**Barrier**	**Challenges**	**Proposed solutions**	**Clinical examples**
Treatment fatigue	Continuous, long-term management exhausts patients both physically and emotionally	Shared decision-making to create flexible, patient-centered care plans	A patient with osteoarthritis feels overwhelmed by physical therapy and medication, disengaging from treatment
	Lack of perceived progress leads to frustration and reduced adherence	Tailor treatment to individual preferences and goals	Incorporating more achievable short-term goals and patient-controlled activities helps the patient regain engagement
	Side effects from long-term pharmacotherapy (e.g., NSAIDs, opioids) further exacerbate fatigue	Use of non-pharmacological options like physical therapy, CBT, or exercise	Introducing cognitive behavioral therapy (CBT) alongside physical therapy to reduce mental and physical burden
Therapeutic inertia	Clinicians hesitate to adjust or escalate treatment, relying on familiar yet suboptimal therapies	Implement multidisciplinary teams for dynamic case discussions and treatment adjustments	A patient with chronic back pain remains on opioid therapy, despite the availability of more effective alternatives
	Fear of side effects or unfamiliarity with new treatments prevents innovation	Encourage clinician education and peer collaboration to build confidence in new therapies	Involving a pain specialist, physiotherapist, and psychologist to evaluate non-opioid alternatives (e.g., duloxetine)
Cognitive dissonance	Patients resist recommended treatments that conflict with their beliefs or experiences	Transparent communication and shared decision-making to align patient expectations with evidence	A patient avoids physical therapy for fear it will worsen their pain, despite recommendations
	Clinicians struggle to change long-standing treatment regimens even when ineffective	Regular reflective clinical practice to help clinicians challenge outdated methods	Clinicians involve patients in understanding how physical therapy can improve pain, reducing resistance
	Decision-making paralysis can lead to treatment delays or avoidance	Use of data-driven tools (e.g., digital feedback from wearables) to provide real-time evidence	Data from wearable devices showing pain improvement with movement encourages patient adherence

Pain management must have a model that focuses on conservative strategies first before the escalation to pharmacological or procedural intervention (14). Treatment reviews must also be included in clinical practice to prevent therapeutic inertia to ensure that the intervention remains appropriate in the long term (10). For practical implementation, PCPs may benefit from structured clinical tools such as the Keele STarT MSK Tool, which stratifies patients into low, medium, or high risk for persistent symptoms and guides care intensity accordingly. Similarly, the British Pain Society's Core Standards for Pain Management Services provides algorithmic guidance on when to escalate from self-management and primary care support to specialist multidisciplinary input. Integrating such tools into clinical software or checklists can support timely decisions and reduce the cognitive burden on busy clinicians. Some PCPs use decision support tools integrated into electronic health records to promote regular review and prevent therapeutic inertia (1, 5, 24). In built algorithms or protocols can also help streamline reviews and reduce cognitive load on the PCPs to make these decisions (20).

PCPs can better overcome treatment fatigue and cognitive dissonance in chronic MSK pain through structured patient education, motivational interviewing, and graduated exposure to exercise (5, 8). These methods enable overcoming resistance to evidence-based practice through the gradual modification of beliefs and facilitating positive behavioral change. This is especially true when patients perceive prior attempts at similar treatments to have not met their expectations. Clinicians should seek to understand and address such underlying patient beliefs and address them through education on chronic pain in general, and how such treatment options can lead to long-term functional improvement and pain relief, whilst also agreeing specific strategies to overcome individual barriers that previously occurred in such treatments. Combining this with motivational interviewing techniques can better empower patients to overcome their resistance and builds a better therapeutic alliance, and has been shown to lead to better treatment adherence in patients with chronic pain (22, 25).

Finally, psychotherapeutic interventions such as cognitive behavioral therapy (CBT), are an excellent addition to chronic MSK pain management when used in combination with ongoing physical therapies, and have shown benefits in reducing pain intensity and improving quality of life and function (26, 27). CBT is useful in addressing maladaptive cognitive and behavioral responses to experiencing chronic pain as well as addressing depressive or anxious responses to chronic pain, such as catastrophic thinking (28). Apart from the demonstrated holistic benefits to patients with chronic MSK pain, we would hypothesize that encouraging patients to engage with a psychological understanding of their pain experience, through methods such as CBT, may better place them to engage with addressing the psychological factors involved in therapeutic inertia or cognitive dissonance, in discussion with their clinicians.

## Conclusion

To better enhance the primary care management of patients with chronic MSK pain, we have identified treatment fatigue, therapeutic inertia, and cognitive dissonance as areas requiring a multi-faceted approach: one that is individualized and patient-centered and incorporates clinical expertise, personalized medicine, multidisciplinary collaboration, and technological innovation.

Primary care physicians are the best positioned to lead this effort by coordinating care across disciplines, and using emerging tools and treatments that enhance patient outcomes. Being able to confront these challenges effecting patients with chronic MSK pain head-on, may lead clinicians to offer better long-term care strategies in the management of chronic MSK pain and improvement in the quality of life of their patients.
